# Impact of rapid antibiotic susceptibility testing for Gram-negative bacteremia varies by pathogen type and resistance: a secondary analysis of the RAPIDS GN trial

**DOI:** 10.1128/spectrum.01789-24

**Published:** 2024-12-16

**Authors:** Ritu Banerjee, Abhigya Giri, Lauren Komarow, Maria Souli, Sarah B. Doernberg, Robin Patel, on behalf of the Antibacterial Resistance Leadership Group

**Affiliations:** 1 Pediatric Infectious Diseases, Vanderbilt University, Nashville, Tennessee, USA; 2 Biostatistics Center, George Washington University, Rockville, Maryland, USA; 3 Duke Clinical Research Institute, Duke University, Durham, North Carolina, USA; 4 Division of Infectious Diseases, University of California, San Francisco, California, USA; 5 Laboratory Medicine and Pathology, Mayo Clinic, Rochester, Minnesota, USA; MultiCare Health System, Tacoma, Washington, USA

**Keywords:** antibiotic resistance, bacteremia, susceptibility testing, bloodstream infections

## Abstract

In this secondary analysis of 386 subjects with Gram-negative bacteremia enrolled in the RAPIDS GN trial, rapid antibiotic susceptibility testing had a greater benefit on the management of bacteremia caused by antibiotic-resistant compared to antibiotic-susceptible isolates, especially for *Escherichia coli, Klebsiella* species*,* and *Proteus* species.

**IMPORTANCE:**

Rapid blood culture diagnostics are costly, and their use has not demonstrated clear clinical benefit for patients with Gram-negative sepsis, possibly because prior trials did not enroll sufficient numbers of patients with antibiotic-resistant infections. This analysis of patients with sepsis previously enrolled in a randomized controlled clinical trial demonstrates that rapid susceptibility testing of bacteria from blood cultures had the greatest impact for patients with antibiotic-resistant infections. Specifically, after rapid testing results were reported, patients infected with resistant bacteria received appropriate antibiotic therapy more quickly than patients with susceptible bacteria. These findings highlight the need to evaluate blood culture diagnostics in areas with high antibiotic resistance where these diagnostics are likely to have the greatest impact.

## OBSERVATION

Patients with bacteremia receive empiric antibiotics before organism identification and antibiotic susceptibility become available. Poor outcomes are associated with antibiotic therapy that is inactive or overly broad spectrum (1–3). Rapid identification of bloodstream pathogens and resistance may lead to earlier pathogen-directed antibiotic treatment.

The RAPIDS GN trial was a prospective multicenter randomized controlled trial demonstrating that rapid phenotypic antibiotic susceptibility testing (AST) directly from positive blood culture bottles led to faster antibiotic therapy changes compared to standard of care (SOC) testing for Gram-negative bacteremia (4). A majority of subjects in the trial had antibiotic-susceptible isolates and underwent antibiotic de-escalation within the first 72 h after randomization. Only a minority of subjects had multi-drug resistant bloodstream isolates that required antibiotic escalation when AST results were available. We performed a secondary analysis of the RAPIDS GN data to assess how pathogen type and antibiotic resistance modified the impact of rapid AST on the appropriateness of antibiotic choice in the first few days after the onset of bacteremia.

We included subjects enrolled in either arm of the RAPIDS GN trial from 2017 to 2018 who had blood cultures with monomicrobial Gram-negative bacilli that were “on-panel” for the rapid test (Accelerate Pheno System) (5), isolated using SOC methods and who received empiric antibiotics. We reviewed clinical and laboratory information stored in electronic files at the trial coordinating center, the Duke Clinical Research Institute. Full methods have been previously described (4).

Antibiotics administered from randomization (0 h) through 52 h were evaluated to enable the capture of therapy changes within the first 48 h after randomization. Antibiotic therapy was classified based on SOC AST as undertreatment (antibiotic to which the blood isolate was resistant), optimal treatment (narrowest spectrum antibiotic to which the blood isolate was susceptible), or overtreatment (overly broad-spectrum antibiotics based on blood isolate AST). Detailed definitions are in the supplemental material. The use of ceftriaxone for ceftriaxone-susceptible *Enterobacter* was considered undertreatment due to concern for inducible *Amp*C. To account for changes in antibiotic-prescribing patterns that may have occurred following the publication of the MERINO 1 and 2 trials (6, 7), two definitions of undertreatment were used (including and excluding piperacillin-tazobactam) for ceftriaxone non-susceptible isolates of *Escherichia coli, Klebsiella* species, and *Proteus* species*,* and all isolates of other Gram-negative, non-*Pseudomonas* species. Antibiotic-resistant isolates were defined as third-generation cephalosporin non-susceptible isolates for *E. coli* or *Klebsiella*, or *Proteus* species, and resistance to one or more of the following for *Acinetobacter, Citrobacter, Enterobacter, Serratia,* and *Pseudomonas* species: cefepime, meropenem, and piperacillin/tazobactam. Ertapenem resistance was also included in the definition of antibiotic-resistant Enterobacterales, but not *Acinetobacter* or *Pseudomonas* species. To compare treatment between groups, the Pearson χ^2^ test was used. A *P*-value < 0.05 was considered statistically significant. All analyses were done in SAS version 9.4.

**Fig 1:**
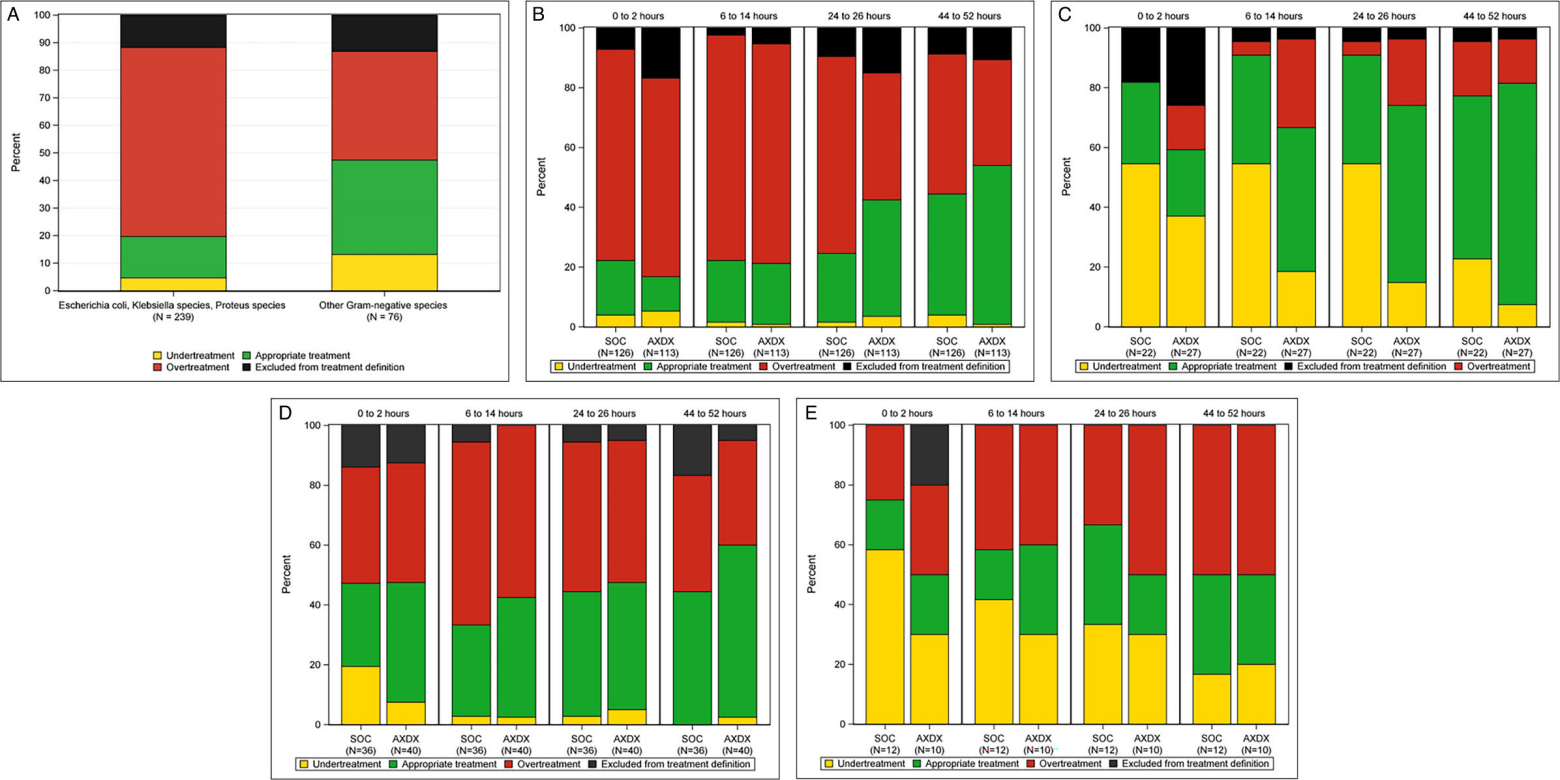
(**A**) Empiric treatment (0–2 h post-randomization) among subjects with antibiotic-susceptible isolates of monomicrobial, on-panel Gram-negative species (*N* = 315). For this analysis, subjects from both SOC and rapid testing groups were included. Black: excluded from treatment definition if subjects received no antibiotics, antibiotics without susceptibility results reported, only an oral cephalosporin or metronidazole, or intravenous or oral azithromycin, penicillin, rifaximin, or atovaquone. (**B**) Antibiotic treatment appropriateness for antibiotic-susceptible *Escherichia coli,* and *Klebsiella* and *Proteus* isolates (*N* = 239) by time after randomization and treatment arm. AXDX, rapid susceptibility testing group. (**C**) Antibiotic treatment appropriateness for antibiotic-resistant (third-generation cephalosporin non-susceptible or carbapenem-resistant) *Escherichia coli,* and *Klebsiella* and *Proteus* isolates (*N* = 49) by time after randomization and treatment arm. (**D**) Antibiotic treatment appropriateness for antibiotic-susceptible *Acinetobacter, Citrobacter, Enterobacter, Serratia,* and *Pseudomonas* isolates (*N* = 76) by time after randomization and treatment arm. (**E**) Antibiotic treatment appropriateness for antibiotic-resistant *Acinetobacter, Citrobacter, Enterobacter, Serratia,* and *Pseudomonas* isolates (*N* = 22) by time after randomization and treatment arm.

We included 386/448 (86%) subjects (196 SOC, 190 rapid testing). Among 315 susceptible isolates, between 0 and 2 h post-randomization (before AST results were available), overtreatment was more common for *E. coli*, *Klebsiella,* and *Proteus* species than for other species (164/239 [69%] vs 30/76 [39%], *P* < 0.001; Fig. 1A). By 52 h, carbapenem use among susceptible isolates was significantly lower in the rapid testing arm compared to the SOC arm (17/68 [18%] vs 12/68 [25%], *P* < 0.001). Among 288 *E. coli, Klebsiella,* and *Proteus* isolates, treatment appropriateness improved with time after randomization in both SOC and rapid testing groups. The proportion with appropriate treatment was higher in the rapid testing group than the SOC group at 24–26 h (60/140 [43%] vs 37/148 [25%], *P* = 0.001) and 48–52 h (80/140 [57%] vs 63/148 [43%], *P* = 0.013) post-randomization. Overtreatment was common for susceptible isolates (Fig. 1B), while undertreatment was common for resistant isolates (Fig. 1C). Among resistant isolates, by 52 h post-randomization, undertreatment decreased to 2/27 (7%) subjects in the rapid testing group and 5/22 (23%) subjects in the SOC group (Fig. 1C). Among 98 subjects with *Acinetobacter, Citrobacter, Enterobacter, Serratia,* and *Pseudomonas* isolates, there was no statistically significant differences in the proportion with appropriate treatment between the rapid testing and SOC arms for susceptible or resistant isolates (Fig. 1D and E). There were no differences when piperacillin-tazobactam was included or excluded in the definition of treatment appropriateness.

In this secondary analysis of the RAPIDS GN trial, we observed that rapid AST had the greatest impact on the antibiotic management of patients with antibiotic-resistant *E. coli, Klebsiella,* or *Proteus* bacteremia, in whom it led to timely antibiotic escalation and less antibiotic undertreatment. In contrast, among subjects with antibiotic-susceptible *E. coli, Klebsiella,* or *Proteus* species, the percentage with antibiotic overtreatment remained high despite the availability of rapid AST results. This may be due to a variety of reasons, including considering antibiotic de-escalation as non-urgent, underestimating the harms of overly broad-spectrum antibiotic use, and unwillingness to stop antibiotics with activity against *Pseudomonas* and *Amp*C-beta lactamase when there is diagnostic uncertainty. Rapid AST also had minimal impact on treatment for subjects with *Acinetobacter, Citrobacter, Enterobacter, Serratia,* or *Pseudomonas* isolates, whether they were antibiotic susceptible or resistant. Future outcome trials evaluating blood culture diagnostics should enroll more subjects with antibiotic-resistant infections to be sufficiently powered to detect clinical outcome differences.

Our findings emphasize that clinicians readily escalate antibiotic therapy but are reluctant to de-escalate treatment despite the availability of AST results, even when coupled with active antibiotic stewardship, as has been described previously (8, 9). The minimal impact of rapid AST on the management of bacteremia caused by Gram-negative species other than *E. coli, Klebsiella*, or *Proteus* may reflect a low sample size. It is also possible that clinicians did not de-escalate antibiotic treatment for subjects with susceptible *Pseudomonas* and/or *Amp*C-producing species because these subjects may have had greater medical complexity than those with *E. coli* or *Klebsiella* bacteremia, although these patient groups did not differ in health characteristics like Charlson Score at enrollment.

This study has limitations. This trial was conducted in the U.S. in sites without a high prevalence of antibiotic resistance, and results may not be generalizable to other geographic areas with higher resistance rates or different antibiotic-prescribing practices. We did not review patient-level clinical data for this analysis, which may have led to misclassification of treatment appropriateness. Regardless, our findings demonstrate that rapid blood culture diagnostics have the greatest clinical impact on therapeutic decisions for antibiotic-resistant bacteremia. These findings highlight the need to implement and evaluate blood culture diagnostics in areas with high antibiotic resistance and for future trials to enroll sufficient numbers of subjects with antibiotic-resistant infections.

## SUPPLEMENTAL MATERIAL

Supplemental material spectrum.01789-24-s0001.docx Treatment definitions.

ASM does not own the copyrights to Supplemental Material that may be linked to, or accessed through, an article. The authors have granted ASM a non-exclusive, world-wide license to publish the Supplemental Material files. Please contact the corresponding author directly for reuse.
