# Supplementary material for: Impact of rapid antibiotic susceptibility testing for Gram-negative bacteremia varies by pathogen type and resistance: a secondary analysis of the RAPIDS GN trial
Source: Microbiol Spectr. 2024 Dec 16;13(2):e01789-24. doi: 10.1128/spectrum.01789-24 (PMC11792449; doi:10.1128/spectrum.01789-24)
Supplement: Supplemental material — Treatment definitions. [file spectrum.01789-24-s0001.docx]

**Impact of rapid antibiotic susceptibility testing for Gram negative bacteremia varies by pathogen type and resistance: A secondary analysis of the RAPIDS GN trial**

**Supplement – Treatment Definitions**

**Abbreviations**

- **C1 = category 1:** ampicillin, cefazolin
- **C2 = category 2:** amoxicillin/clavulanate, ampicillin/sulbactam, ceftriaxone, cefotaxime, ciprofloxacin, levofloxacin, trimethoprim-sulfamethoxazole
- **C3 = category 3:** ceftazidime
- **C4 = category 4:** aztreonam, cefepime, ertapenem*, piperacillin/tazobactam
- **C5 = category 5:** meropenem, ceftazidime/avibactam, ceftolozane/tazobactam, colistin, polymyxin B, tigecycline
- **GP = gram positive drugs:** clindamycin, daptomycin, linezolid, tedizolid, and vancomycin
- **AM = aminoglycosides:** amikacin, gentamicin, tobramycin
- **Drugs excluded from treatment definitions:** atovaquone, azithromycin, cephalosporin (Oral), metronidazole, Penicillin VK
- **R/I =** resistant or intermediate susceptibility
- **S =** susceptible

*****Ertapenem-resistance not included in definitions of resistant *Acinetobacter* and *Pseudomonas* species, which are intrinsically resistant to this drug.

***E. coli, Klebsiella,* and *Proteus* species**

Ceftriaxone-susceptible isolates

*Appropriate treatment*

- Receipt of only one drug from C1 or C2 to which the isolate tested susceptible
- Receipt of AM plus an appropriate drug (as defined above) is considered appropriate

*Undertreatment*

- Receipt of any C1, C2, C3, or C4 drug or meropenem if isolate tested R/I to the drug used
- Receipt of monotherapy with either AM, GP, or doxycycline
- Receipt of AM, GP or doxycycline with other antibiotics that are considered undertreatment

*Overtreatment*

- Receipt of one drug from C1 or C2 to which isolated tested susceptible, and receipt of any other drug that is not AM
- Receipt of any C5 drug other than meropenem
- Receipt of any C3 or C4 drug or meropenem if isolate tested susceptible to the drug used

Ceftriaxone-resistant isolates

*Appropriate treatment*

- PRE-MERINO^1^: Receipt of only one of the following drugs to which the isolate tested susceptible: ciprofloxacin, levofloxacin, trimethoprim-sulfamethoxazole, piperacillin/tazobactam, ertapenem, meropenem.
- POST-MERINO: Receipt of only one of the following drugs to which the isolate tested susceptible: ciprofloxacin, levofloxacin, trimethoprim-sulfamethoxazole, ertapenem, meropenem.
- Note: Co-administration of AM with an appropriate drug (as defined above) is considered appropriate.

*Undertreatment:*

- PRE-MERINO
  - Receipt of any of following: C1 drugs, amoxicillin/clavulanate, ampicillin/sulbactam, ceftriaxone, cefotaxime, aztreonam, cefepime, or ceftazidime
  - Receipt of a drug to which the isolate tested R/I: ciprofloxacin, levofloxacin, trimethoprim-sulfamethoxazole, piperacillin/tazobactam, ertapenem or meropenem
  - Receipt of monotherapy with AM, GP or doxycycline
  - Receipt of AM, GP or doxycycline with other antibiotics that are considered undertreatment
- POST-MERINO
  - Receipt of any of following: C1 drugs, amoxicillin/clavulanate, ampicillin/sulbactam, ceftriaxone, cefotaxime, aztreonam, cefepime, ceftazidime or piperacillin/tazobactam
  - Receipt of a drug to which the isolate tested R/I: ciprofloxacin, levofloxacin, trimethoprim-sulfamethoxazole, ertapenem or meropenem
  - Receipt of monotherapy with AM, GP or doxycycline
  - Receipt of AM, GP or doxycycline with other antibiotics that are considered undertreatment

*Overtreatment:*

- PRE-MERINO
  - Receipt of one of the following drugs to which the isolate tested susceptible: ciprofloxacin, levofloxacin, trimethoprim-sulfamethoxazole, piperacillin/tazobactam, ertapenem, meropenem, AND receipt of another drug that is not an AM
  - Receipt of any C5 drug that is not meropenem, if isolate is not resistant to meropenem or ertapenem
- POST-MERINO
  - Receipt of one of the following drugs to which the isolate tested susceptible: ciprofloxacin, levofloxacin, trimethoprim-sulfamethoxazole, ertapenem, meropenem, AND receipt of another drug that is not an AM
  - Receipt of any C5 drug that is not meropenem, if isolate is not resistant to meropenem or ertapenem

***Acinetobacter, Citrobacter, Enterobacter, Serratia* species**

*Appropriate treatment:*

- PRE-MERINO-2^2^
  - Receipt of only one of the following drugs to which isolate tested susceptible: cefepime, ciprofloxacin, levofloxacin, trimethoprim-sulfamethoxazole, piperacillin/tazobactam
  - If R/I or not tested to cefepime or piperacillin/tazobactam, then:
    - Receipt of ertapenem only if susceptible to ertapenem (except for *Acinetobacter* species)
    - Receipt of meropenem only if susceptible to meropenem
    - Receipt of one C5 drug that is not meropenem if meropenem and ertapenem resistant
- POST-MERINO-2: Same considerations as above except piperacillin/tazobactam is not considered appropriate, even if isolate is susceptible to it
- Note: Co-administration of AM with an appropriate drug (as defined above) is considered appropriate.

*Undertreatment:*

- PRE-MERINO-2
  - Receipt of C1, C3, or aztreonam
  - Receipt of some C2 drugs: amoxicillin/clavulanate, ampicillin/sulbactam, ceftriaxone, cefotaxime
  - Receipt of other drugs to which isolate tested R/I: cefepime, ciprofloxacin, levofloxacin, trimethoprim-sulfamethoxazole, piperacillin/tazobactam
  - Receipt of ertapenem if isolated tested R/I for ertapenem
  - Receipt of Meropenem if isolated tested R/I for Meropenem
- POST-MERINO-2
  - Same considerations as above
  - Receipt of piperacillin/tazobactam
  - Receipt of ertapenem for *Acinetobacter* species

*Overtreatment:*

- PRE-MERINO-2:
  - Receipt of one of the following drugs to which isolate tested susceptible: cefepime, ciprofloxacin, levofloxacin, trimethoprim-sulfamethoxazole, or piperacillin/tazobactam, AND receipt of another non-AM drug
  - Receipt of ertapenem or meropenem when susceptible to one or more the following drugs: cefepime, ciprofloxacin, levofloxacin, trimethoprim-sulfamethoxazole, piperacillin/tazobactam
  - If R/I to all of the above drugs in the list (or R/I to cefepime and R/I or not tested to the other drugs in the list), then:
    - Receipt of more than one C5 drug that is not meropenem when resistant to both ertapenem and meropenem
    - Receipt of one or more C5 drugs that is not meropenem when susceptible to meropenem or ertapenem
- POST-MERINO-2:
  - Receipt of one of the following drugs to which isolate tested susceptible: cefepime, ciprofloxacin, levofloxacin, trimethoprim-sulfamethoxazole, AND receipt of another non-AM drug
  - Receipt of ertapenem or meropenem when susceptible to one or more the following drugs: cefepime, ciprofloxacin, levofloxacin, trimethoprim-sulfamethoxazole
  - If R/I to all of the above drugs in the list (or R/I to cefepime and R/I or not tested to the other drugs in the list), then:
    - Receipt of more than one C5 drug that is not meropenem when resistant to both ertapenem and meropenem
    - Receipt of one or more C5 drugs that is not meropenem when susceptible to meropenem or ertapenem

***Pseudomonas* species**

*Appropriate treatment:*

- Receipt of one of the following drugs to which isolate tested susceptible: cefepime, ceftazidime, ciprofloxacin, levofloxacin, piperacillin/tazobactam, aztreonam
- If R/I or not tested to cefepime or piperacillin/tazobactam, then:
  - Receipt of meropenem if isolate is susceptible to meropenem
  - Receipt of one C5 drug that is not meropenem if meropenem-resistant
- Receipt of AM with the other appropriate drugs (as defined above) is considered appropriate

*Undertreatment*

- Receipt of C1 drugs
- Receipt of the following C2 drugs: amoxicillin/clavulanate, ampicillin/sulbactam, ceftriaxone, cefotaxime, trimethoprim-sulfamethoxazole
- Receipt of ertapenem
- Receipt of other drugs to which isolate tested R/I: aztreonam, ciprofloxacin, levofloxacin, meropenem, piperacillin/tazobactam, cefepime or ceftazidime

*Overtreatment*

- Receipt of non-AM drugs with appropriate drugs (as defined above)
- Receipt of C5 drugs other than meropenem for isolates that tested susceptible to meropenem
- Receipt of more than one C5 drug that is not meropenem if isolate tested R/I to cefepime, ceftazidime, ciprofloxacin, levofloxacin, piperacillin/tazobactam, aztreonam or meropenem

**References**

1. Harris PNA, Tambyah PA, Lye DC, MERINO Trial Investigators and the Australasian Society for Infectious Disease Clinical Research Network (ASID-CRN). [Effect of Piperacillin-Tazobactam vs Meropenem on 30-Day Mortality for Patients With *E coli* or *Klebsiella pneumoniae* Bloodstream Infection and Ceftriaxone Resistance: A Randomized Clinical Trial.](https://pubmed-ncbi-nlm-nih-gov.proxy.library.vanderbilt.edu/30208454/) JAMA. **2018**; 320(10):984-994.
2. Stewart AG, Paterson DL, Young B, MERINO Trial Investigators and the Australasian Society for Infectious Disease Clinical Research Network (ASID-CRN). [Meropenem Versus Piperacillin-Tazobactam for Definitive Treatment of Bloodstream Infections Caused by AmpC beta-Lactamase-Producing *Enterobacter* spp, *Citrobacter freundii*, *Morganella morganii, Providencia* spp, or *Serratia marcescens*: A Pilot Multicenter Trial (MERINO-2).](https://pubmed-ncbi-nlm-nih-gov.proxy.library.vanderbilt.edu/34395716/)Open Forum Infect Dis. **2021;** 8(8):ofab387.
